# Investigation on the Microstructure and Mechanical Properties of X70 Pipeline Steel Fabricated by Laser-Directed Energy Deposition

**DOI:** 10.3390/ma18214997

**Published:** 2025-10-31

**Authors:** Zhandong Wang, Chunke Wang, Linzhong Wu, Guifang Sun

**Affiliations:** 1 College of Mechanical and Electronic Engineering, Nanjing Forestry University, Nanjing 210037, China; ck_wang@njfu.edu.cn (C.W.); 3240300248@njfu.edu.cn (L.W.); 2 School of Mechanical Engineering, Southeast University, Nanjing 211189, China

**Keywords:** pipeline steel, additive manufacturing, directed energy deposition, thermal process, microstructure, tensile strength

## Abstract

The laser-directed energy deposition (L-DED) technique, with its excellent environmental adaptability and superior repair capability, shows great potential for the repair of damaged X70 pipeline steel. In this work, the microstructure and mechanical properties of L-DED repaired X70 steel were systematically investigated. The deposited material exhibited inhomogeneity along the building direction. From the bottom to the top, the grains gradually coarsened, and the proportion of polygonal ferrite increased. This was mainly attributed to increasing thermal accumulation with deposition height, which reduced the cooling rate and promoted solid-state transformations at higher temperatures. Meanwhile, the heat accumulation and intrinsic heat treatment reduced the dislocation density and promoted Fe_3_C precipitation within grains and along boundaries. Microhardness was highest in the bottom region and decreased along the building direction due to the gradual coarsening of microstructure and decreasing in dislocation density. The L-DED X70 showed lower yield strength (435 MPa) and ultimate tensile strength (513 MPa) compared to the base material and API 5L requirements. The elongation of the L-DED X70 was 42.9%, which was 58% higher than that of the base material, indicating excellent ductility. These results revealed a thermal history-dependent strength–ductility trade-off in the L-DED repaired X70 steel. Therefore, more efforts are needed to control the L-DED thermal process, tailor the microstructure, enhance strength, and meet the service requirements of harsh environments.

## 1. Introduction

X70 pipeline steel, characterized by excellent strength-toughness balance and favorable weldability, has become a critical material for long-distance transportation of energy sources such as oil and natural gas [1]. However, during prolonged service, pipelines are frequently exposed to harsh operating conditions involving complex stress states, corrosive environments, and coupled multiphase media, rendering their surfaces highly susceptible to damage such as cracking, pitting corrosion, and mechanical wear [2,3]. Leakage incidents not only endanger human safety and cause environmental pollution but also result in substantial economic losses. Therefore, the development of efficient and reliable on-site repair technologies is of significant engineering importance for ensuring the safe operation of pipeline networks and extending their service life.

Arc welding repair is currently the most widely adopted technique. However, its high heat input tends to induce microstructural coarsening and residual stress, making it difficult to meet the quality requirements of high-standard engineering applications. In recent years, laser-directed energy deposition (L-DED) technology has gained widespread application in aerospace, automotive, and marine industries [4,5]. L-DED exhibits many advantages, such as low heat input, narrow heat-affected zone, and minimal thermal distortion [6]. In addition, rapid laser melting and solidification effectively suppress grain growth, yielding a dense, fine-grained microstructure that enhances the overall mechanical performance of the repaired zone [7]. Thus, investigating the applicability of L-DED technology for repairing damaged X70 pipeline steel holds considerable scientific value and engineering potential.

The L-DED melt pool solidifies under non-equilibrium, rapid solidification conditions, resulting in a unique solidification microstructure within the repaired region. As the repaired zone is constructed via sequential track-by-track and layer-by-layer metal deposition, thermal cycling further modifies the characteristics of the solidified microstructure. Cheng et al. [8] demonstrated that the extremely high cooling rate inherent to L-DED significantly suppresses austenite grain growth and promotes the formation of fine-grained transformation products such as bainite and martensite, in contrast to the coarse columnar grains typically observed in conventional welding. Rashid et al. [9] investigated the microstructural features of additively manufactured 300 M ultra-high-strength steel. They demonstrated that the deposited zone consists primarily of untempered martensite, while overlapping scan tracks, subjected to additional thermal cycles, develop tempered martensite. Ning et al. [10] pointed out that the microstructure of X80 pipeline steel fabricated by the wire-fed additive manufacturing technique is dominated by lath bainite (LB). The grain size and phase composition are distinctly different from those produced by conventional methods. These results are also reported by Seede et al. [11]. Collectively, these studies indicate that the additive manufacturing process parameters and solidification characteristics jointly govern the microstructural constitution of the repaired zone, revealing fundamental differences compared to traditional techniques.

The unique microstructure of the L-DED repaired zone profoundly influences its mechanical properties. Sun et al. [12] applied laser cladding to repair AISI 4340 high-strength steel. They claimed that while the repaired zone exhibited markedly elevated microhardness, its tensile properties were significantly degraded. Zhan et al. [13] reported that the thermal cycling in the repair layer of AerMet100 ultra-high-strength steel induced grain refinement, leading to substantially higher microhardness and strength than the substrate, albeit with a pronounced reduction in ductility. Similar results are reported by Jing et al. [14]. Sun et al. [15] investigated the L-DED repair of HSLA-100 high-strength steel. They found that the complex thermal histories result in non-uniform microhardness distribution. These findings demonstrate that the heat input, composition, and cooling path modulate the microstructure, thereby profoundly affecting the mechanical response of the repaired zone. Despite abundant research on L-DED of high-strength steels, systematic investigations specifically targeting damage repair of X70 pipeline steel remain lacking, particularly concerning the mechanisms of microstructural evolution in the repaired zone and its quantitative correlation with mechanical properties.

To the best of our knowledge, powder-blown L-DED is firstly applied to repair the damaged X70 pipeline steel. The effects of thermal cycles and heat accumulation during L-DED on the microstructure, microhardness, and tensile properties of the repaired region are systematically investigated. Through comprehensive comparison with rolled base material (BM), the underlying mechanisms by which the L-DED process affects microstructural evolution and mechanical properties of the pipeline steel are thoroughly elucidated. This work aims to provide a scientific foundation for high-performance repair of X70 pipeline steel by L-DED.

## 2. Experimental Procedure

### 2.1. Materials

The BM is standard X70 pipeline steel supplied by Wuyang Iron and Steel Co., Ltd. (Pingdingshan, China). This steel plate is produced by the thermo-mechanical control process (TMCP). The dimensions of the prepared BM are 200 mm × 100 mm × 14 mm (length × width × height). The additively manufactured sample (AM) was fabricated using the X70 powder. The X70 powder was produced from the as-received X70 steel plates via the gas atomization technique. The particle size distribution of the X70 powder ranges from 45 to 105 μm. The chemical composition of the X70 plate and X70 powder is listed in Table 1. The chemical composition of the steel plate was analyzed using optical emission spectroscopy (SPECTRO LMF15; SPECTRO Analytical Instruments GmbH, Kleve, Germany). The chemical composition of the powder was measured using inductively coupled plasma optical emission spectroscopy (Spectro ICP-OES, SPECTRO Analytical Instruments GmbH, Kleve, Germany), while the contents of the non-metallic elements C and S were determined using an inert gas fusion analytical instrument (NCS CS-2800; Luoyang Shibida Precision Instruments Co., Ltd., Luoyang, China).

### 2.2. L-DED Process

**Figure 1:**
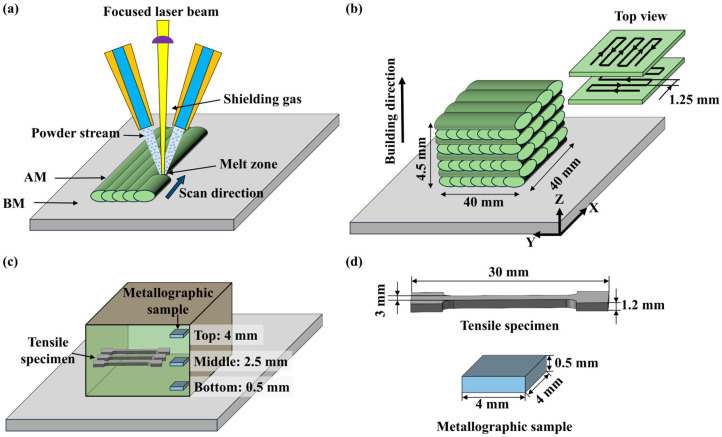
(**a**) L-DED process, (**b**) scan strategy, (**c**) sampling location, and (**d**) dimension of the tested samples.

The L-DED process was performed on a laser cladding forming system (AFS-C1280, Beijing Longyuan AFS Co., Ltd., Beijing, China). This system primarily consisted of the following modules: a fiber-coupled laser device (Trudisk 6002, Trumpf, Ditzingen, Germany), a three-axis numerically-controlled machine tool, and a coaxial powder-feeding laser cladding head (T003, Nanjing Huirui Photoelectric Technology Co., Ltd., Nanjing, China). The laser beam was delivered via a 600 μm core-diameter optical fiber to the laser cladding head and focused onto the substrate surface with a focused spot diameter of 2 mm. The laser cladding head was precisely controlled by the CNC machine tool to execute spatial trajectory movements, synchronously feeding X70 powder into the melt pool. The L-DED process is illustrated in Figure 1a.

As shown in Figure 1b, the L-DED scan path employed a layered reciprocating scanning strategy, with the dimensions of the formed sample being 40 mm × 40 mm × 4.5 mm (length × width × height). To prevent the oxidation of the melt pool during L-DED, high-purity nitrogen was used as the shielding gas at a flow rate of 18 L/min. Based on the results of preliminary process exploration, optimized process parameters were selected for the repair experiment, with specific parameters detailed in Table 2. This work utilized the energy volume density (EVD) to characterize the heat input resulting from the process parameters. It was calculated using Equation (1) [16]:

(1) $$EVD=\frac{P}{v\cdot d\cdot h}$$

where P is the laser power, v is the scanning speed, d is the diameter of laser spot, h is the layer height. Based on the measurement result of the single track, the average height of the single track was 1.2 mm. Substituting the corresponding parameters into the equation yielded a EVD value of 50 J/mm^3^.

### 2.3. Microstructure Characterization

The sampling locations for the metallographic specimens are shown in Figure 1c, with specific dimensions and shapes detailed in Figure 1d. Samples were taken from the top, middle, and bottom regions to comparatively analyze the microstructural characteristics at different deposition heights. The specimen surfaces were sequentially mechanically ground using SiC abrasive papers of 80, 240, 600, 1000, and 2000 grit, followed by fine polishing with 2.5 μm diamond polishing compound. A 4% nitric acid alcohol solution was used for chemical etching to reveal the microstructure. After etching, the microstructure was examined using optical microscopy (OM; Primotech, Carl Zeiss, Oberkochen, Germany) and scanning electron microscopy (SEM; Nova Nano, FEI Company, Hillsboro, OR, USA).

The internal grain structure was further examined using transmission electron microscopy (TEM; Tecnai G2, FEI Company, Hillsboro, OR, USA). The twin-jet electrolytic polishing method was employed, using an electrolyte composed of a mixture of 10 vol.% perchloric acid (HClO_4_) and 90 vol.% ethanol (C_2_H_5_OH), to thin the samples until they became electron beam transparent. Thin areas of the TEM samples were further used for electron backscatter diffraction analysis (EBSD; Oxford Instruments, Abingdon, UK). The EBSD scanning field of view was 250 × 190 μm^2^, with a sampling step size of 0.36 μm. The AZtecCrystal V2.1 software was used for analyzing the obtained EBSD data.

### 2.4. Microhardness and Tensile Testing

Microhardness testing was performed using a microhardness tester (HXD-1000 TMSC/LCD, Shanghai Taiming Optical Instrument Co., Ltd., Shanghai, China). The applied load was 200 gf, with a dwell time of 15 s. For the AM specimen, microhardness measurements were initiated from the topmost surface and conducted at intervals of 0.5 mm along the depth direction. Total ten measurement points were used for the analysis. Each set of measurements was repeated for three times, and the average value was taken to minimize experimental error. For the BM specimen, ten measurement points were randomly selected at different locations. The average microhardness value was calculated for comparative analysis with the microhardness distribution of the AM specimen.

Tensile specimens were sampled as indicated in Figure 1c, with specific dimensions and geometry shown in Figure 1d. Tensile specimens were extracted from the middle region of the AM block to represent the characteristic mechanical behavior under stable deposition conditions. The initial layers had high cooling rates, while the last layers experienced severe heat accumulation. Both conditions led to non-uniform microstructures and localized property variations. In contrast, the middle region experienced relatively stable thermal cycles, resulting in a more representative and homogeneous microstructure that reflected the steady-state condition of the process.

The tensile specimens were mounted on an electronic universal testing machine (NKK-4050, Shenzhen Nanfang Jingke Instrument & Equipment Co., Ltd., Shenzhen, China) for quasi-static uniaxial tensile testing at a strain rate of 0.3 mm/min. Each test was repeated three times. The average values were used to ensure data reliability. The ultimate tensile strength (UTS) and post-fracture elongation of both AM and BM specimens were experimentally determined. Due to the absence of an extensometer, the yield strength (YS) could not be measured directly. Instead, it was estimated from the engineering stress–displacement curve using the deviation analysis method. The yield strength was determined as the stress corresponding to a 5% deviation from the linear elastic behavior predicted by Hooke’s law [17]. In addition, the fracture surfaces of the tensile specimens were analyzed by SEM.

## 3. Results

### 3.1. Microstructure

**Figure 2:**
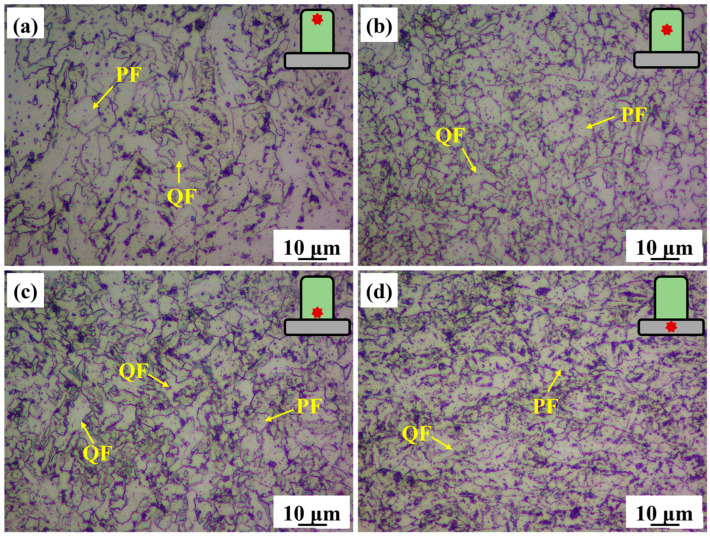
OM images showing the microstructural features of the X70 specimens. (**a**) Top region of AM specimen, (**b**) middle region of AM specimen, (**c**) bottom region of AM specimen, and (**d**) BM specimen.

Figure 2 shows the microstructural characteristics of the AM and BM X70 specimens. Figure 2a–c show that the microstructure of the AM specimen is primarily composed of polygonal ferrite (PF) and quasi-polygonal ferrite (QF). However, the grain size and phase fraction varied significantly across different regions. Specifically, the top region of the AM specimen exhibited coarse microstructure which was predominantly composed of large PF (Figure 2a). In the middle region, the grain size was refined. PF was more frequently observed than QF (Figure 2b). In contrast, the bottom region contained a higher proportion of QF compared to PF (Figure 2c). Figure 2d shows that the microstructure of the BM specimen is predominantly composed of QF. QF tended to exhibit a flattened morphology resulting from the rolling process during TMCP. In addition, the grain size of the BM specimen was smaller than that of the AM specimen.

**Figure 3:**
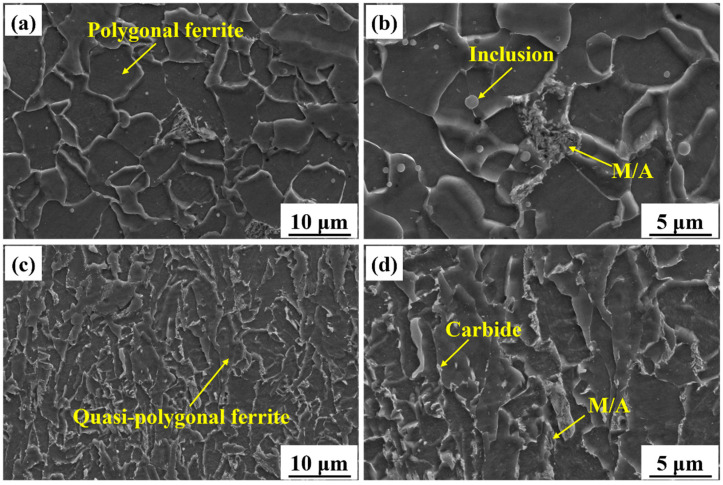
SEM images showing the typical microstructures of the X70 specimens. (**a**,**b**) Middle region of AM specimen and (**c**,**d**) BM specimen.

For a concise and clear comparison between the AM and BM microstructures, the microstructure from the middle region of the AM specimen was selected for detailed analysis. The SEM images in Figure 3 showed the microstructures of the AM and BM specimens. Figure 3a reveals that the microstructure of the AM specimen is predominantly composed of regularly shaped PF, exhibiting equiaxed or near-equiaxed morphology. PF grain boundaries are clearly defined. Figure 3b clearly shows that the M/A island-like constituents are located along grain boundaries and distributed between PF grains. In addition, numerous spherical inclusions were dispersed within the grains. In contrast, Figure 3c shows that the microstructure of the BM specimen is primarily composed of QF, with smaller, irregularly shaped grains and wavy grain boundaries. Figure 3d exhibits the carbides and small M/A constituents along the grain boundaries. The M/A constituents were predominantly located at grain boundary regions.

**Figure 4:**
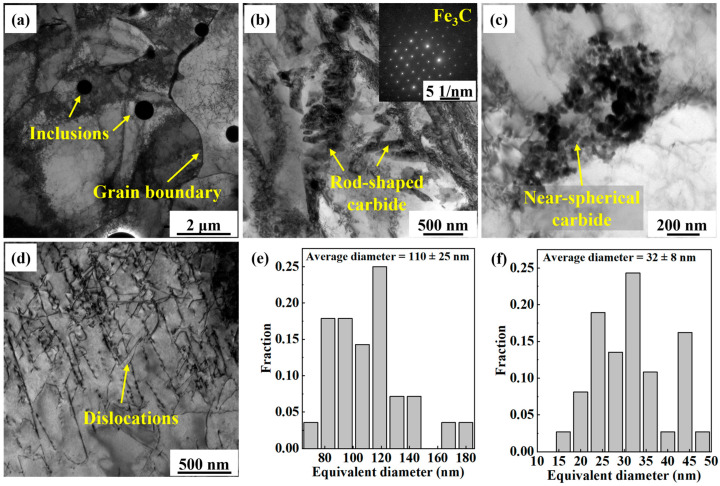
TEM bright-field images showing the microstructure in the middle region of AM X70 specimen. (**a**) Low-magnification of microstructure, (**b**) rod-shaped carbide aggregation, (**c**) near-spherical carbide aggregation, (**d**) sparse and straight dislocation lines, (**e**) size distribution of the rod-shaped carbides in (**b**), and (**f**) size distribution of the near-spherical carbides in (**c**).

Figure 4 presents the TEM analysis results of the middle region of the AM specimen. Figure 4a shows that dispersed inclusions are distributed within the PF grains. Grain boundaries between adjacent grains were clearly discernible. Figure 4b,c show the high-magnification images of the grain interiors, further revealing the distribution characteristics of carbides. Figure 4b shows that numerous rod-shaped carbides are interconnected and locally clustered within the grains. Selected area electron diffraction (SAED) analysis verified that the precipitated carbide was cementite (Fe_3_C). Figure 4c shows that many near-spherical carbides are enriched along grain boundaries, exhibiting pronounced segregation behavior. Figure 4e,f compare the size distributions of the carbides with different shapes. The average diameter of the rod-shaped carbides (110 ± 25 nm) was much larger than that of the near-spherical carbides (32 ± 8 nm). Figure 4d shows that the dislocation lines in the AM X70 specimen are straight and sparsely distributed. This suggests minimal plastic strain accumulation during the deposition process, resulting in a significantly low dislocation density, which is consistent with the characteristics of PF as a diffusion-controlled transformation product.

**Figure 5:**
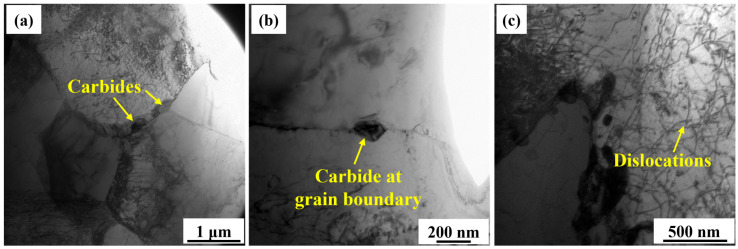
TEM bright-field images showing the microstructure of the BM X70 specimen. (**a**) Low-magnification of microstructure, (**b**) carbide distributed at the grain boundary, and (**c**) entangled and intersecting dislocations.

Figure 5 presents the TEM analysis results of the BM specimen. Figure 5a shows a low-magnification image of the BM microstructure. Carbides precipitated at the trigeminal grain boundaries. In addition, some dislocation lines were distributed in the grains. Figure 5b shows that the carbides precipitate at the straight grain boundaries of two grains. The morphology and distribution of the carbides in the BM specimen were distinctly different from those of the AM specimen (Figure 4b,c). Figure 5c shows that the interior of the BM grains contains a dense network of entangled dislocations, with dislocation density notably higher than that in the AM specimen (Figure 4d). Dislocation density was even higher in grain boundary regions, forming typical dislocation pile-up structures, as shown in Figure 5a.

**Figure 6:**
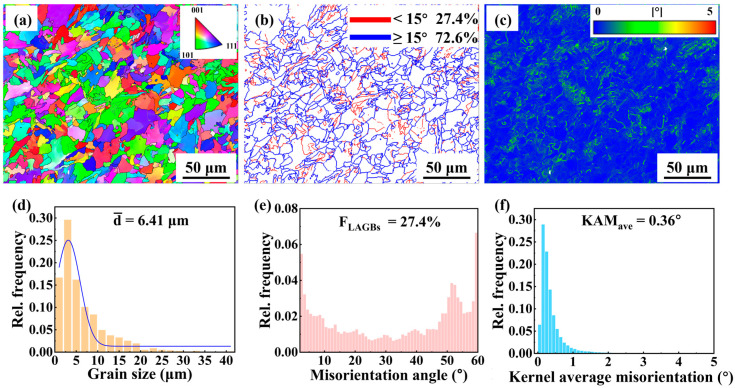
EBSD maps of the middle region of the AM specimen. (**a**) IPF map, (**b**) grain boundary distribution map, (**c**) KAM map, (**d**) distribution histogram of grain size, (**e**) distribution histogram of grain boundary misorientation angle, and (**f**) distribution histogram of KAM.

The EBSD analysis results of the middle region of the AM specimen are shown in Figure 6. The inverse pole figure (IPF) in Figure 6a revealed a random crystallographic orientation distribution. The grain size distribution in Figure 6d showed an average grain size of approximately 6.41 μm, with most grains smaller than 20 μm and a few grains reaching up to 40 μm. The grain boundary map in Figure 6b showed that the high-angle grain boundaries (HAGBs, ≥15°) accounted for 72.6% of total boundaries, while the low-angle grain boundaries (LAGBs, 2–15°) constituted 27.4%. The fractions of the HAGBs and LAGBs are further illustrated in Figure 6e. The HAGBs were predominantly located at PF boundaries, whereas the LAGBs were mainly distributed within PF grains. The kernel average misorientation (KAM) map in Figure 6c showed that the local misorientation was confined to a few grains, suggesting limited localized plastic strain accumulation. The distribution of the local misorientation agreed well with the distribution of LAGBs (Figure 6b). The KAM histogram in Figure 6f showed an average value of ~0.36°, with most values below 1.5°. The average density of the geometrically necessary dislocations (GND) can be calculated by the equation $\rho_{GND}=\frac{2{KAM}_{ave}}{\mu b}$, where μ is the step size of EBSD experiment, and b is the length of the Burgers vector. The $\rho_{GND}$ of the AM specimen was (1.89 ± 1.64) × 10^14^ m^−2^.

**Figure 7:**
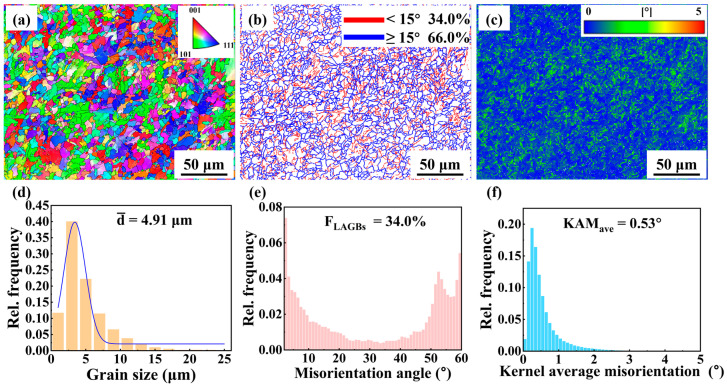
EBSD maps of the BM specimen. (**a**) IPF map, (**b**) grain boundary distribution map, (**c**) KAM map, (**d**) distribution histogram of grain size, (**e**) distribution histogram of grain boundary misorientation angle, and (**f**) distribution histogram of KAM.

The EBSD analysis results of the BM specimen are shown in Figure 7. Figure 7a shows that no obvious preferred orientation is observed in the BM specimen. The grain size distribution in Figure 7d showed an average grain size of 4.91 μm. The grain size of BM specimen was smaller than that of the AM specimen. The fractions of HAGBs and LAGBs were 66% and 34%, respectively, as shown in Figure 7b. The fraction of HAGBs in the BM specimen was lower than that in the AM specimen (Figure 6b). Figure 7e shows that the misorientation angle distribution of LAGBs peaks around 2°, indicating that they are primarily formed by dislocation lines. The KAM map in Figure 7c showed that the distribution of local misorientation in the BM specimen was relatively uniform. The KAM histogram in Figure 7f showed an average value of ~0.53° which was higher than that in the AM specimen (0.36°), indicating that the local strain in the BM specimen was relatively high. The $\rho_{GND}$ of the BM specimen was (2.56 ± 1.83) × 10^14^ m^−2^ which was higher than that of the AM specimen.

### 3.2. Microhardness

**Figure 8:**
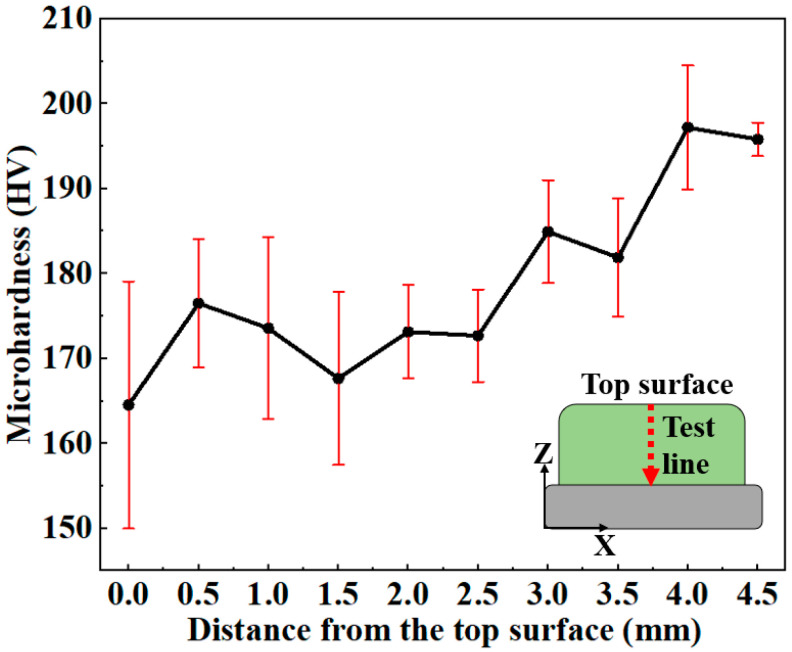
Microhardness distribution along the depth direction of the AM X70 specimen.

Figure 8 shows the microhardness distribution along the depth direction of the AM specimen. The microhardness of the top region was the lowest, while that of the bottom region was the highest. The microhardness gradually increased along the depth direction, which was consistent with the microstructural heterogeneity through the building direction (Figure 2a–c). The average microhardness of the AM specimen was calculated as 179 ± 11 HV. The average microhardness of the BM specimen was 235 ± 7 HV. The microhardness of the AM specimen is 23.8% lower than that of the BM specimen, attributed to microstructural differences such as grain size and dislocation density.

### 3.3. Tensile Properties

**Figure 9:**
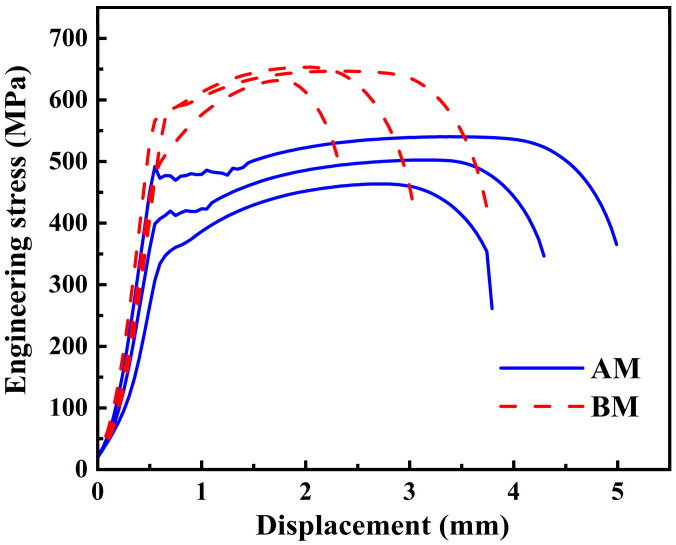
Engineering stress-displacement curves of the AM and BM specimens.

Figure 9 shows the engineering stress-displacement curves of the AM and BM specimens. The tensile specimens were taken from the middle region of the AM block, as shown in Figure 1c. The corresponding tensile test results are listed in Table 3. The AM specimen exhibited a clear yield plateau, indicating the presence of a yield point phenomenon. The BM specimen exhibited no distinct yield plateau. Due to the absence of an extensometer, the work hardening behavior beyond the yield point cannot be quantified. However, a qualitative comparison of the engineering stress-displacement curves of the AM and BM specimens revealed that the AM specimen exhibited a lower degree of work hardening than the BM specimen. Compared to the AM specimen, the BM specimen exhibited higher YS and UTS but lower elongation. According to the API 5L specification standard for X70 steel [18], the specified minimum YS is 485 MPa, and the minimum UTS is 570 MPa. However, the measured YS of the AM specimen was 434.8 MPa, which was quite below the required 485 MPa, indicating that the AM X70 did not meet the API 5L standard. This is mainly due to the formation of soft PF in the as-deposited microstructure.

**Figure 10:**
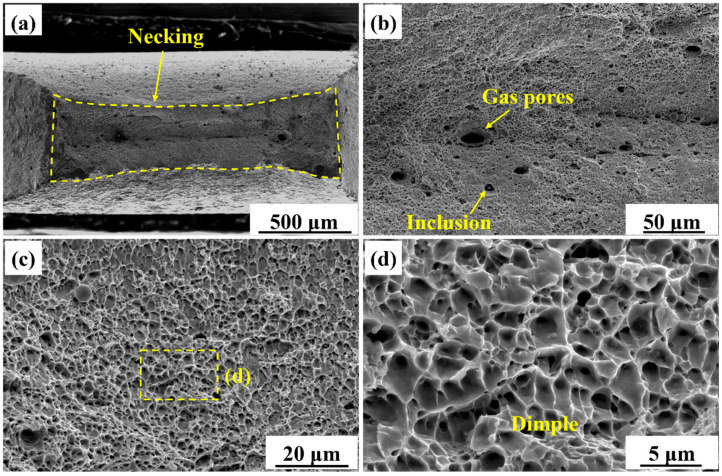
Fracture morphology of the AM tensile specimen. (**a**) Macroscopic morphology, (**b**) gas pores and inclusions, (**c**) uniformly distributed dimples, (**d**) magnified view of the dimples.

Figure 10 shows the tensile fracture morphology of the AM specimen. Figure 10a reveals a significant necking area, with a measured reduction in area of 84.2% ± 2.7%. This indicated a significant plastic deformation during the fracture process of the AM specimen. Figure 10b reveals the presence of gas pores, inclusions and dimples on the fracture surface, demonstrating a ductile fracture of the AM specimen. The interface between the inclusions and the matrix was weak, making the interface prone to form micro-voids around them under tensile loading. Figure 10c,d show magnified views of the dimples. The uniform distribution and significant depth of the dimples were consistent with the microvoid coalescence (MVC) mechanism. The dimple sizes were concentrated in the 2–4 μm range.

**Figure 11:**
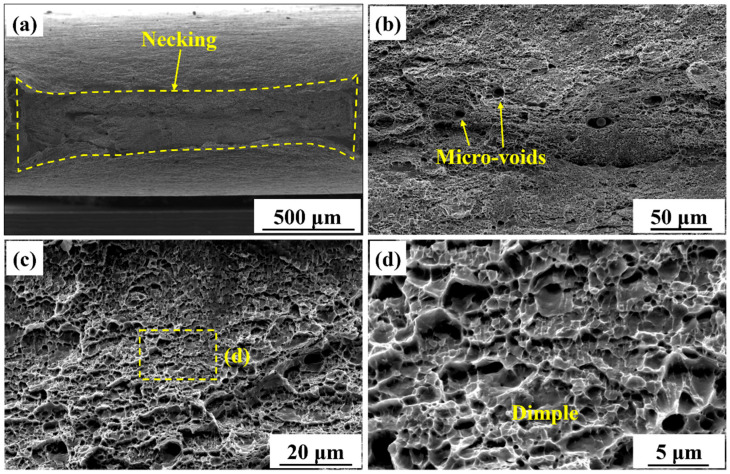
Fracture morphology of the BM tensile specimen. (**a**) Macroscopic morphology, (**b**) micro-voids on the surface, (**c**) uneven distribution of dimples, and (**d**) magnified view of dimples.

Figure 11 shows the tensile fracture morphology of the BM specimen. Figure 11a shows significant necking in the BM specimen. The measured reduction in area was 83.4% ± 3.5%, which was similar to that of the AM specimen. This indicated that both specimens exhibited comparable necking behavior. Figure 11b reveals the presence of micro-voids and dimples on the fracture surface. The formation, growth and coalescence of micro-voids were typical mechanisms of ductile fracture. Figure 11c,d show the magnified views of the dimples. The dimples in the BM specimen, which varied in size, were irregularly distributed. Figure 11d reveals shallower dimples compared to those in the AM specimen, indicating a lower degree of plastic deformation and inhibited micro-void expansion. The plastic deformation capacity of the BM specimen was lower than that of the AM specimen.

## 4. Discussion

### 4.1. Influence of L-DED Process on Microstructure Evolution

Experimental results revealed significant differences in the microstructures of the AM and BM specimens, particularly in grain morphology, size, and carbide precipitation. These microstructural variations directly influence the strength and ductility of the materials. The fundamental cause of these microstructural differences lies in the distinct thermal histories experienced during processing, with cooling rate and thermal cycling being key determinants of the microstructural evolution pathway [19].

#### 4.1.1. Thermal Process During L-DED

In L-DED, a high-energy laser beam was used to generate a melt pool on the substrate or previously deposited layers, into which metal powder was simultaneously injected. Three-dimensional components were fabricated through successive layer-by-layer deposition. This layer-wise buildup imparted a complex thermal history to the material. Regions within the deposited layers underwent multiple thermal cycles, each involving rapid heating to a peak temperature followed by cooling [20].

During the early stages of L-DED, heat was primarily conducted into the substrate. The low initial substrate temperature created a steep thermal gradient between the melt pool and the substrate, facilitating efficient heat extraction. As the deposition height increased, heat accumulated in the deposited material. Subsequent heat must pass through the previously deposited layers before reaching the substrate. These layers were at elevated temperatures, which reduced the overall thermal gradient and impaired the heat dissipation efficiency, resulting in progressively lower cooling rates for subsequent layers [21]. The above-mentioned thermal process significantly influenced the formation and evolution of microstructures.

#### 4.1.2. Phase Transformation of L-DED X70 Steel

**Figure 12:**
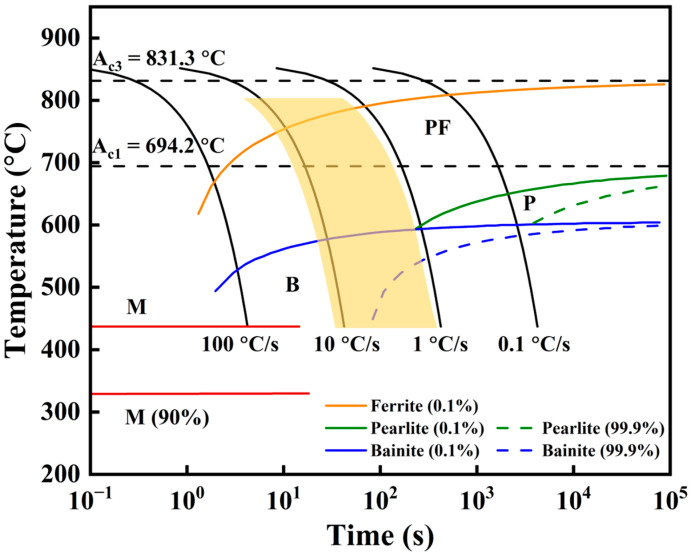
CCT curve of the X70 pipeline steel. The translucent region refers to the range of cooling curves in this work corresponding to the transformation from austenite to ferrite.

It is well established that the cooling time from 800 °C to 500 °C (t_8/5_) is a critical factor determining the microstructure formed during solid-state transformation in low-carbon steels [22]. The CCT curve of X70 steel was calculated by Jmatpro V7.0 software, as shown in Figure 12. During continuous deposition, the cooling rates range from approximately 2 to 20 °C/s [23]. According to the CCT curve of the X70 steel (Figure 12), these cooling rates favored the formation of PF [24,25]. However, different cooling rates and transformation temperature ranges lead to the formation of different types of ferrite. In the first deposited layer, the low substrate temperature resulted in a relatively high cooling rate, shifting austenite-to-ferrite transformation to a lower temperature range (600–680 °C). The solid-state transformation occurring within this temperature range was favorable for the formation of QF (Figure 2c). Figure 1b shows that the deposited block is relatively small. As the deposition height increased, heat accumulation within the build became increasingly significant. The temperature range for the transformation gradually increased. In the middle region, the moderate cooling rate promoted the formation of PF, resulting in an increased proportion of this phase (Figure 2b). At the top, the temperature range for the transformation was the highest (650–750 °C), coupled with the slowest cooling rate. This allowed sufficient time for the microstructure to fully grow, resulting in the formation of coarse PF and QF (Figure 2a).

Related studies have also pointed out that the cooling rates during AM significantly affect the formation of the deposited microstructure. Ning et al. [10] demonstrated that the microstructure of the wire-fed AM X80 pipeline steel consists of LB due to the low heat input and rapid cooling rate. With increasing cooling rate, LB is preferentially formed. The formation of LB is accompanied by a shift in the transformation mechanism from a diffusion-controlled process to a mixed-mode mechanism involving both shear and diffusional characteristics. Wang et al. [26] pointed out that the cooling time t_8_/_5_ governs the continuous cooling transformation behavior of undercooled austenite. Compared to other heat affected zones in the X100 pipeline steel, the t_8_/_5_ of the laser-welded zone is the shortest (0.6 s), resulting in the formation of microstructure consisting of lath martensite and large bulk bainitic ferrite. As can be seen from the above comparison, the microstructure of the pipeline steel is primarily dependent on the cooling rate. PF tends to form under relatively slow cooling conditions.

EBSD results show that the grain size of the AM specimen is larger than that of the BM specimen. This difference arose from their distinct processing routes and thermal histories. During the layer-by-layer deposition, heat accumulation occurred progressively. This thermal buildup promoted repeated thermal cycling, leading to gradual coarsening of the microstructure into coarse PF. No external plastic deformation was applied to the as-deposited block, except for the mechanical constraints imposed by the substrate [27]. Therefore, the solid-state transformation and microstructural evolution were primarily governed by the high-temperature thermal cycles during L-DED. In contrast, the TMCP for X70 steel involved controlled rolling followed by accelerated cooling. As a result, the final microstructure was dominated by QF, characterized by a flattened morphology induced by plastic deformation and a refined grain structure due to fast cooling [28,29]. The differences in processing routes and thermal histories lead to significant variations in microstructure and mechanical properties.

#### 4.1.3. Carbide Precipitation

TEM results showed that there were two types of carbides formed in the AM X70, as shown in Figure 4b,c. The rod-shaped carbides in Figure 4b were mainly distributed along the grain boundary. During the diffusional phase transformation from austenite to PF, carbon atoms were expelled to the austenite grain boundaries [30,31]. In addition, the quite large heat accumulation and slow cooling rates provided sufficient driving force for carbon atom diffusion and Fe_3_C nucleation and growth [23]. The intrinsic heat treatment during L-DED is somewhat like the tempering treatment used in the production routes of steel [32]. The main difference between this intrinsic heat treatment and conventional tempering is that the heating and cooling processes of the deposited layers are relatively fast. The thermal cycles promoted the precipitation of Fe_3_C. In contrast, conventional tempering treatments involve longer durations and higher temperatures, under which M_23_C_6_ generally precipitates from the microstructure and aggregates at grain boundaries [33]. The M_23_C_6_ ((Cr, Mn, Fe)_23_C_6_) exhibits higher stability than the M_3_C ((Cr, Mn, Fe)_3_C) [34].

The near-spherical carbides in Figure 4c were concentrated in a small region. These carbides were transformed from the M/A constituents formed during the cooling process. During the solid-state phase transformation process, the carbon atoms were ejected from the ferrite and partitioned into austenite. The carbon can improve the stability of the austenite. Further cooling led to the transformation of part of the austenite into martensite. As a result, a small amount of M/A constituents was retained in the microstructure [35]. The intrinsic heat treatment promoted the decomposition of the M/A constituents, resulting in the precipitation of theses carbides.

#### 4.1.4. Grain Boundary and Dislocation Evolution

EBSD analysis revealed that the proportion of HAGBs in the AM specimen was 72.6%, significantly higher than that in the BM specimen (66.0%). This difference can be attributed to the distinct thermal cycles experienced during AM and TMCP. When the deposited layers were subjected to high temperatures (above A_c3_ or between A_c3_ and A_c1_), grain growth and recrystallization occur, modifying the as-deposited microstructure. When the temperature was below A_c1_, in situ tempering occurred [23]. In these processes, small grains merged into large grains, leading to the formation of HAGBs. Meanwhile, the diffusional phase transformation from austenite to PF were not accompanied by the formation of dislocations. The pre-existed dislocations underwent rearrangement and annihilation when they were exposed to the thermal cycles [36], leading to the reduction in dislocation density. As a result, the fraction of LAGBs was reduced in the AM specimen. In contrast, the rolling and controlled cooling processes during TMCP resulted in the formation of a large number of subgrain boundaries and dislocations in the microstructure. This significantly increased the fraction of the LAGBs in the BM specimen.

### 4.2. Influence of Microstructure on Microhardness

The AM specimen exhibited lower microhardness than the BM specimen. Compared with the BM specimen, the coarse PF grains and low dislocation density in the AM specimen contributed to its reduced microhardness. In addition, the AM specimen exhibited a pronounced microhardness gradient along the building direction. This microhardness gradient was determined by the spatially varying microstructural features. The microstructure in the bottom region was dominated by QF. QF formed through a mixed mechanism of diffusion and shear [24]. Its grain boundaries were slightly curved and uneven. It contained some dislocation tangles and subgrains inside. Therefore, the QF in the bottom region had higher microhardness. The middle region had a higher content of PF. Fe_3_C precipitates were presented within the ferrite grains. The interstitial solid solution strengthening effect of carbon atoms was significantly reduced due to carbon partitioning into precipitated carbides. As a result, the microhardness of the middle region was decreased. The coarse microstructure in the top region contributed to the reduction in microhardness. In contrast, the BM specimen possessed a fine-grained microstructure and a high dislocation density, both of which significantly increased the resistance to dislocation motion, leading to higher deformation resistance [37,38].

Table 4 summarizes the microhardness of the X70 pipeline steel fabricated by different methods. The microhardness of the arc-welded X70 is lower than the standard, whereas that of the laser-welded X70 is higher [39,40,41]. The microhardness is influenced by the microstructure, which is determined by the cooling rate. The microhardness distribution of the AM specimen is well below the standard, reflecting the low strengthening capability of the PF-dominated microstructure.

### 4.3. Influence of Microstructure on Tensile Properties

#### 4.3.1. Tensile Strength and Ductility

The YS and UTS of the AM specimen were significantly lower than those of the BM specimen. According to the Hall-Petch relationship, the YS of the metallic material is inversely proportional to the square root of its grain size [42]. EBSD analysis revealed that the AM specimen was dominated by coarse PF and exhibits a low grain boundary density, as shown in Figure 6a,b. In addition, the TEM and EBSD results were consistent in showing that the AM specimen had a low dislocation density. The L-DED X70 exhibited limited strengthening capability due to its coarse microstructure and low dislocation density [43].

As shown in Figure 9, the AM specimen exhibited excellent ductility, which was closely related to its internal microstructure. The AM specimen contained a high proportion of HAGBs. These HAGBs can deflect the propagation direction of cracks within the material, causing them to consume more energy during propagation, thereby effectively inhibiting crack growth and enhancing the ductility [44]. Additionally, HAGBs facilitated dislocation transmission across grain boundaries, promoting a more uniform strain distribution throughout the polycrystalline structure and delaying localized necking.

Furthermore, the initial dislocation density in the AM specimen was relatively low, allowing dislocations to have higher mobility during the early stages of plastic deformation [45]. This not only alleviated the premature pile-up of dislocations at grain boundaries [46], which hindered dislocation movement, but also reduced stress concentrations that typically triggered micro-void nucleation, further enhancing the ductility.

#### 4.3.2. Fracture Morphology

The fracture surface of the AM specimen exhibited typical ductile fracture characteristics, as shown in Figure 10. The coarse PF matrix, high fraction of HAGBs, and low dislocation density collectively contributed to a more homogeneous plastic strain distribution. Micro-void nucleation occurred predominantly at inclusions and second-phase particles. Subsequently, they underwent stable growth and coalescence under uniform stress conditions. As a result, deep dimples were formed through the MVC mechanism. In contrast, the dimple morphology in the BM specimen was shallow and non-uniform. The BM specimen consisted of fine grains and exhibited a high dislocation density. The BM specimen had high strength but limited ductility, leading to poorly developed dimples, as shown in Figure 11d.

### 4.4. Limitations

The L-DED process offers notable advantages for repairing X70 pipeline steel, including in situ repair capability, controllable heat input, adjustable powder composition, and fast repair speed. However, this study also reveals certain limitations. Significant heat accumulation and slow cooling rates associated with L-DED lead to the formation of PF. This deteriorates the mechanical properties of the as-deposited X70 steel. The tensile strength does not meet the mechanical property requirements specified in the API 5L standard [18]. It is widely reported that the ideal microstructure for X70 steel is AF, which exhibits superior mechanical properties, including high strength and toughness [47,48]. However, AF formation typically requires higher cooling rates. To optimize the mechanical properties of the deposited X70, future research could focus on controlling thermal cycles and cooling profiles to locally enhance the cooling rate, thereby promoting AF formation.

Potential technical solutions include: (i) Adjust the interlayer residence time. Heat accumulation can be reduced by extending the interlayer cooling time [49]. The next layer begins to deposit on a substrate at a lower temperature. (ii) Near-immersion active cooling. The build platform is lowered to bring the deposited layer into contact with a cooling medium, thereby enhancing heat dissipation [50]. (iii) Spray cooling. A spray cooling system can be integrated into the L-DED process for interlayer temperature control [51]. The cooling medium can be high-pressure air or liquid nitrogen. These strategies will be applied to L-DED X70 pipeline steel to improve its mechanical properties through controlled microstructure development.

## 5. Conclusions

This study employed the L-DED technique to repair the X70 pipeline steel. The microstructural characteristics and mechanical properties of the L-DED X70 steel were systematically investigated and compared with those of the BM. The influence mechanisms of the L-DED process on the comprehensive performance of the AM X70 were elucidated. The main conclusions are as follows:

(1) The microstructure exhibited inhomogeneity along the building direction. From the bottom to the top, the grains gradually coarsened, and the proportion of PF increased. From the bottom to the top, thermal accumulation increased, resulting in decreased cooling rates, which promoted grain coarsening and the formation of PF. The grain size of the AM X70 was larger than that of BM.
(2) AM X70 exhibited a significantly reduced dislocation density. The high thermal input and slow cooling rates led to substantial annihilation of dislocation structures. Fe_3_C carbides were dispersed both within grains and along grain boundaries. Meanwhile, the intrinsic heat treatment provided favorable kinetic conditions for carbon diffusion, as well as for the nucleation and growth of Fe_3_C.
(3) The microhardness gradually decreased along the building direction. The bottom region had a high content of QF, resulting in higher microhardness. In contrast, the top region was predominantly composed of coarse PF, leading to lower microhardness. The average microhardness of the AM specimen was 179 ± 11 HV which was 23.8% lower than that of the BM specimen (235 ± 7 HV).
(4) The AM X70 exhibited a YS of 435 MPa and an UTS of 513 MPa. Both the YS and UTS of the AM X70 were lower than those of the BM and API 5L standard. The elongation of AM X70 reached 42.9%, which was 58% higher than that of the BM. The reduction in strength stemmed from the grain coarsening and decreased dislocation density induced by the L-DED thermal cycles. Meanwhile, the plastic accommodation ability was enhanced, resulting in substantially improved ductility. In harsh service environments characterized by complex stress states, the strength of repaired pipeline steel becomes critically important. Given the current limitations in mechanical performance, future work will primarily focus on enhancing the strength of the deposited X70 steel.

## Figures and Tables

**Table 1:** Chemical composition (wt.%) of the X70 plate and X70 powder.

Element	C	Mn	Ni	Cr	Si	Mo	V	Cu	Nb	P	S	Fe
X70 plate	0.085	1.57	0.02	0.27	0.21	0.003	0.003	0.024	0.05	0.010	0.005	Bal.
X70 powder	0.057	1.47	0.08	0.36	0.20	0.013	0.002	0.02	0.04	0.012	0.008	Bal.

**Table 2:** L-DED process parameters.

Parameter	Value
Laser power (W)	2000
Laser spot diameter (mm)	2
Scanning speed (mm/min)	1000
Powder feed rate (g/min)	10
Hatching distance (mm)	1.25
ΔZ increment (mm)	0.75
Layer number	8

**Table 3:** Summary of the tensile test results of the AM and BM specimens.

Material	Yield Strength (MPa)	Ultimate Tensile Strength (MPa)	Elongation (%)
X70 AM	434.8 ± 36.4	512.6 ± 24.2	42.9 ± 3.5
X70 BM	549.7 ± 46.4	643.7 ± 10.9	27.1 ± 7.1
X70 API 5L	≥485	≥570	≥20

**Table 4:** Summary of the microhardness of X70 pipeline steel fabricated by different methods.

Method	Microhardness (HV)	Refs.
Submerged arc welding	220–240	[39]
Submerged arc welding	206–220	[40]
Laser welding	370–380	[41]
API 5L	240–280	[18]
L-DED	165–197	This work

## Data Availability

The original contributions presented in this study are included in the article. Further inquiries can be directed to the corresponding authors.
